# Immuno-histochemical evaluation of CD34 for OLP and OSMF

**DOI:** 10.6026/973206300200358

**Published:** 2024-04-30

**Authors:** Harshit Shrivastava, Mahesh Shenoy, Nishath Sayed Abdul, Deepashree Pramod Gujjar, Ajoy Kumar Shahi, Preeti Dhir, Bhumika J Patel, Ramanpal Sing Makkad

**Affiliations:** 1 Department of Oral and Maxillofacial Surgery, Hitkarini Dental College and Hospital, Jabalpur, Madhya Pradesh., India; 2 Department of OMFS & Diagnostic Sciences, College of Medicine & Dentistry, Riyadh Elm University, P.O. Box 84891 - Riyadh 11681, Kingdom of Saudi Arabia; 3 Faculty of Oral Pathology, Department of OMFS and Diagnostic Sciences, College of Medicine and Dentistry, Riyadh Elm University, Riyadh, Kingdom of Saudi Arabia; 4 Department of Oral pathology and Microbiology, Bapuji dental college and hospital, Davanagere-544007, Karnataka, India; 5 Department of Oral & Maxillofacial Surgery, Dental Institute, Rajendra institute of Medical Sciences, Ranchi, India; 6 Department of Oral Medicine and Radiology, Dasmesh Institute of Research and Dental Sciences, Faridkot, Punjab, India; 7 Department of Dentistry, Howard University, College of Dentistry, Washington DC, USA; 8 Department of Oral Medicine and Radiology, New Horizon Dental College and Research Institute, Bilaspur, Chhattisgarh, India

**Keywords:** CD34, immuno-histochemistry, OLP, OSMF

## Abstract

Immuno-histochemical evaluation of CD34 in oral lichen planus (OLP) and Oral Submucous Fibrosis (OSMF) is of interest to dentist.20 specimens of normal oral mucosa (buccal mucosa/gingiva tissue) from patients who had extractions performed as part of orthodontic treatment comprised Group I, the control group. Group II comprised 30 individuals with a diagnosis of oral lichen planus. 30 OSMF cases with diagnoses is Group III. These 80 specimens were all given consideration when choosing for CD34immuno-histochemical staining. The CD34 was greater in all categories of OLP and OSMF when compared to normal controls. Maximum CD34 expression was observed in erosive OLP (147.41±17.60) followed by OSMF (116.01 ±11.72) and reticular OLP (105.01±11.62). Data was statistically significant (p<0.001).Immunohistochemistry of CD34 evaluation is a potential diagnostic marker for OLP and OSMF.

## Background:

The mouth and throat is believed to represent the overall health or illness of the body, acting as a means of early detection [1, 2-3]. Oral manifestation frequently precedes additional symptoms or abnormalities at other sites and coexists with a variety of systemic disorders OLP is a prevalent mucocutaneous condition that was first reported by Wilson in 1869 [4, 5, 6]. Its estimated global prevalence is 0.89% [7, 8, 9]. Kaposi reported the first clinical variety of LP in 1892, while Wickham documented the reticular white line's properties. Darier is attributed with providing the first comprehensive interpretation of the histological quirks associated with LP [8- 10].Numerous factors have been identified as potential causes, such as stress, heredity, systemic disorders, infections, dental restorative materials, and medications [11-12]. On the other hand, research points to oral lichen planus (OLP) as an autoimmune condition mediated by T cells, whereby cytotoxic CD8+ T-cells trigger the death of oral epithelial cells [13, 14, 15]. It's unclear what exactly caused it [4- 6]. Among the most often impacted areas are the nails, scalp mucous membranes of the mouth and genitalia and skin. The buccal and labial mucosa is among the most prevalent locations in the mouth cavity [4- 8]. The six clinical types of OLP include bullous, atrophic, erosive, plaque, reticular and popular. The clinical presentation varies from subclinical white keratotic plaques to excruciating erosions including ulcerations [6-8]. The chronic, sneaky oral potentially malignant condition (OPMD) known as oral submucous fibrosis (OSMF) results in fibrosis of tissue, the deposition of collagen and the creation of scar tissue [7-9]. OSMF is widely recognized as an Asian illness, with a particular emphasis on the Indian subcontinent because of the region's high areca nut combined tobacco usage [12, 16, 17]. Progressive OSMF deteriorates oral health and makes it harder to speak and chew. Furthermore, oral squamous cell carcinoma (OSCC), with transformation into cancer rates that vary from 1.5 to 15%, is a possibility for those with OSMF [12- 16]. The malignant transformation processes for OSMF are distinct from those of other OPMDs [15]. This discrepancy may be connected to particular areca nut carcinogenic qualities. Specifically, areca alkaloids cause cytotoxic and genotoxic effects on the oral epithelium [16]. Another possibility is that collagen accumulation in the submucosa might lead to tissue hypoxia, which is a cancer-inducing factor [17]. Pro-inflammatory cytokines are elevated and anti-fibrotic IFN-gamma is decreased when areca nut consumption results in chronic irritation. This leads to increased stiffness and juxta-epithelial-pro-inflammatory reaction, which ultimately causes epithelial degeneration [18, 19, 20, 21]. Consequently, a number of molecular processes in the connective tissue and epithelium result in the development of malignant forms of OSMF; for this reason, a great deal of research has been done on location-specific biomarkers in OSMF [22, 23, 24, 25]. In the basal as well as parabasal layers of the epithelium, Bax, Bcl2, Ki-67 and P53 expression are said to be highly expressed in OSMF and are considered to be early indicators of the malignant progression of OSMF [19-22]. It is commonly known that angiogenesis contributes to the pathophysiology of chronic inflammatory diseases [4- 8]. It boosts the intricate mechanism of feedback and recycling of the cells that regulates the process of inflammatory responses in addition to increasing oxygenation and metabolites to the proliferative tissue [9- 14]. Micro-vessel density (MVD) evaluation has been found to be a highly reliable prognostic marker for a number of tumor types. A study reports that CD34 expression evaluation to be a beneficial angiogenic promoter in lichen planus and many other PMD [16-20]. Because CD34 monoclonal antibodies have the ability to stain vascular endothelial cells, they can be utilized to highlight micro vessels in neoplastic and inflammatory diseases like OLP and OSMF [12-14]. Therefore, immuno-histochemical evaluation of CD34 for OLP and OSMF is of interest.

## Materials and Method:

Eighty patients were included and divided into four groups.

## Criteria for exclusion:

Individuals who smoke or chew tobacco, as well as those who have diabetes

## Qualifications for inclusion:

Individuals with OLP and OSMF diagnose both clinically and histologically. Twenty specimens of normal oral mucosa (buccal mucosa/gingiva tissue) from patients who had extractions performed as part of orthodontic treatment made comprised Group I, the control group. Group II comprised thirty individuals with a diagnosis of oral lichen planus. Thirty OSMF cases with diagnoses make up Group III. These 80 specimens were all given consideration when choosing for CD34immunohistochemical staining.

## Immunohistochemistry:

Sections of tissue, with a thickness of 4-5 µm, were placed on glass slides that were electrostatically charged. After that, the slides were placed in a hot air oven set at 60° for one hour to ensure that the parts adhered properly. The slides were deparaffinized using two changes of xylene for ten minutes each, and they were subsequently rehydrated by immersing them in progressively lowering alcohol concentrations (from 100% to 50%) for five minutes each. In order to achieve antigen retrieval, specimens submerged in Tris-ethylenediaminetetraacetic acid (pH 9) buffer were heated for five minutes to restore pH equilibrium. Using a microwave antigen retrieval apparatus, the tissue was immersed in AR1 solution for two cycles in order to reveal the cytoplasmic antigenic sites in the tissue sections.

In the very first cycle, the average temperature was 90° for 10 minutes, and in the second, it was 98° for 15 minutes, with an instant cooling period in between. The slides were cleaned three times, one minute apart, using phosphate buffer solution (PBS), which has a pH between 7.2 and 7.6. After cleaning the slides, a 10-minute application of peroxide blocking reagent was made. After smearing the slides with power block solution for ten minutes at room temperature to decrease background staining and avoid nonspecific binding, the slides were incubated for one hour at room temperature with a primary mouse monoclonal antibody from tissue culture supernatant diluted in PBS for CD34.

After three PBS washes, the sections were incubated in the super-enhancer solution for twenty minutes. After 30 minutes of room temperature incubation with the polymer horseradish peroxidase reagent, the sections were subjected to three separate 3-minute buffer washes. The slices were then dehydrated using ethanol and xylene, treated for five minutes with a freshly made substrate called diaminobenzidine, counterstained using Harris hematoxylin, and mounted using Digital Picture Exchange.

## Evaluation of the density of micro-vessels:

The sections of OLP OSMF and normal oral mucosal tissue were immuno-stained using anti-CD34 monoclonal antibody to color the micro-vessels. A single vessel was defined as any endothelial cell, either alone or in a cluster that had brown staining and was clearly separated from neighboring micro-vessels, histiocytes, and various other connective tissue components. In order to identify the most vascular locations (hot spot regions) for CD34 that was virtually exclusively confined within the inflammatory infiltrate the stained regions were initially examined at low power (x10). Photographs under x40 were taken of up to five fields in order to count the total amount of blood vessels located in hot spot regions.

## Statistical analysis:

After data entry, the mean ± standard deviation was displayed. SPSS version 21 software for statistical analysis was used to analyze the unpaired t-test, one-way analysis of variance, and Tukey's multiple comparison tests. A P < 0.05 statistically significant value was considered significant.

## Results:

The CD34 was greater in all categories of OLP and OSMF when compared against the normal controls. Maximum CD4 expression was observed in erosive OLP (147.41±17.60) followed by OSMF (116.01 ±11.72) and reticular OLP (105.01±11.62). The findings were significant statistically (p <0.001) (Table 1). There was increased CD4 in both males and females of all study groups of reticular OLP, erosive OLP and OSMF when compared to normal controls. The CD4 MVD in males was comparable to females in all groups (p>0.05) (Table 2).

## Discussion:

Quantification of microvasculature can be done by assessment of mean MVD. In the current study, CD34 was used for the assessment of MVD. CD34 are the cell surface 110-120 (Kilodalton) KD monomeric transmembrane glycoprotein and pan-endothelial markers of endothelial cells. CD34 is considered to be highly sensitive for endothelial cells and produces minimum background staining [4- 6]. Hence, in this present study, CD34 was used for the evaluation of MVD. Our study showed that CD 34 was greater in all categories of OLP and OSMF when compared against the normal controls. Maximum CD4 expression was observed in erosive OLP (147.41±17.60) followed by OSMF (116.01 ±11.72) and reticular OLP (105.01±11.62). The findings were significant statistically (p <0.001).The results of the current study are consistent with other studies [4, 11, 12]. Numerous studies have focused on the etiology and pathophysiology of OLP, and a number of antigen-specific along with nonspecific inflammatory pathways have been proposed to clarify the pathophysiology. It may be inferred from the current study that angiogenesis constitutes one of the reasons that cause the succession of reticular and erosive OLP and OSMF because it was found to be much higher in these conditions than in normal tissue.

The prospect of OLP and OSMF becoming malignantly has been the subject of intense discussion for more than a century; the erosive variation and OSMF is more prevalent in this regard [14-17]. It might be proposed that one of the contributing factors to the elevated malignant potential of erosive OLP and OSMF is the increase in MVD when compared to the reticular form. Although the precise the cause of illness of oral lymphopoiesis remains unknown, auto-cytotoxic CD8+T cells are thought to play a distinct role in inducing apoptosis in the basal cells layer of the mouth epithelium [21-24]. It is believed that the angiogenic phenomenon is crucial to numerous processes that are both physiological as well as pathological [22- 25]. The stimulation of endothelial cells brought on by cytokine release and ischemia or hypoxia sets off the angiogenic response. Since OLP is an inflammatory autoimmune condition, it satisfies every requirement for hypoxia [17-24]. Angiogenesis is a phenomenon that is linked to a number of angiogenic molecules, including transforming growth factor, TNFα, and vascular endothelial growth factor (VEGF). A number of markers, including CD31, CD34, and CD105, are used to assess blood vessel density [19- 24].

Our study epitomizes a direct valuation of the existence of angiogenic events in OLP since it is based on scrutiny through histopathological samples of oral mucosa. Our work, which is based on examination of oral mucosa histopathology samples, embodies a direct appraisal of the presence of angiogenic processes in OLP. A study elevated VEGF marker expression in the erosive as well as reticular pattern [16-21]. According to a recent meta-analysis, 1.1% of OLP lesions progress to OSCC. OLP's malignant transformation may be dependent upon or connected to a number of molecular triggers that cause an inflammatory infiltration [21-25]. A small number of chemicals and radicals produced by inflammation have the ability to affect cell cycle regulators, including cell cycle arrest, intercellular adhesion molecules, and apoptosis [15-17]. The majority of OLP cases have demonstrated elevated COX-2 expression. COX-2 has a number of roles, including interfering with arachidonic acid metabolism, promoting angiogenesis, suppressing the immune system, and blocking apoptosis [14- 16].

It is well known that angiogenesis contributes significantly to inflammation and is a feature of many chronic inflammatory lesions [14-17]. A direct link between angiogenesis and the pathophysiology of OLP has not been demonstrated in several investigations. The use of antiangiogenic medications in combination with traditional OLP treatment may prove advantageous in mitigating the patient's reliance on corticosteroids [18- 21].

## Conclusion:

Immunohistochemical CD4 evaluation can be used as diagnostic marker for OLP and OSMF.

## Figures and Tables

**Table 1:** Evaluation of CD34 between different categories

	**Reticular pattern**	**Erosive pattern**	**OSMF**	**Normal (control)**
Minimum	76	122	87	26
Maximum	124	176	135	54
Mean±SD	105.01±11.62	147.41±17.60	116.01 ±11.72	44.15±9.81
ANOVA (P)		<0.001		

**Table 2:** Evaluation of CD 34 between different categories and genders

	**Reticular pattern**	**Erosive pattern**	**OSMF**	**Normal (control)**
Male (Mean± SD)	109.11±10.74	149.61±9.11	119.10±10.74	45.90±5.41
Female (Mean± SD)	105.21±11.02	145.12± 22.07	115.11 ±10.91	41.13±10.38
P value	0.4939	0.7599	0.4939	0.4284
